# Colica Pictonum from the Medical Employment of Acetate of Lead

**Published:** 1852-03

**Authors:** 


*Colica Pictonum from the medical employment of Acetate of Lead.*—Dr. L. S. Joynes reports (Stethoscope, Dec. 1851) a case of colica pictonum, in a gentleman, æt. 25, who had suffered for several years from chronic diarrhea, and to whom Dr. J. gave acetate of lead and opium. This arrested for the time the diarrhea, but two weeks afterwards the patient was attacked severely with colica pictonum. Only thirty grains of the lead had been taken during the four days it was employed. The most perfect crystals of the salt were selected, more than enough distilled vinegar was given with it to neutralize any of the carbonate, and after each dose a teaspoonful of vinegar was taken.

Cases of this kind are rare, but Dr. J. refers to several; one mentioned by Dr. Burton, where the patient took fifteen grains in five days, and experienced severe colic. Prof. Trousseau, in his work on Therapeutics, quotes a case in which the patient took six grains of the neutral acetate of lead daily for three successive days; the fourth day, a most violent saturnine colic supervened, with jaundice, constipation, retraction of the belly, etc., which only yielded to the "treatment of La Charité," energetically employed. The most remarkable case of all is given by

Devergie, in his *Méd. Legalé :* A student of medicine consulted Professor Fouquier, who prescribed for him some pills containing each one grain of acetate of lead, of which he was directed to take one every day. The *first pill* produced slight colic, the second acted more severely, and the third occasioned such violent symptoms that some mistake of the apothecary was suspected. The pills were analyzed by Devergie, but were found to contain nothing but acetate of lead.

It may be urged, and with truth, Dr. J. remarks, that such cases are exceptional, and that they may be explained by the existence of idiosyncrasies in the subjects of them, such as are known to exist with respect to mercury, opium, and other remedies. But, allowing this to be true, it must also be admitted that a prudent physician is bound to regard such idiosyncrasies in his practice. The knowledge of their occasional existence should be a warning against the use of hazardous remedies, except under circumstances imperatively calling for their employment. —*Ibid.*

